# Supplementary material for: Protein corona formed on lipid nanoparticles compromises delivery efficiency of mRNA cargo
Source: Nat Commun. 2025 Sep 30;16:8699. doi: 10.1038/s41467-025-63726-2 (PMC12485112; doi:10.1038/s41467-025-63726-2)
Supplement: Supplementary file 1 — Supplementary Information File [file 41467_2025_63726_MOESM1_ESM.pdf]

Supplementary Information

Protein corona formed on lipid nanoparticles compromises delivery efficiency of mRNA cargo

Elizabeth Voke[1], Mariah L. Arral[2], Henry J. Squire[1], Teng-Jui Lin[1], Lining Zheng[1], Roxana Coreas[1], Alison Lui[1], Anthony T. Iavarone [3], Rebecca L. Pinals*[4,5], Kathryn A. Whitehead*[2,6], and Markita P. Landry*[1, 3, 7]

*Co-corresponding authors

[1]Department of Chemical and Biomolecular Engineering, University of California, Berkeley, Berkeley, CA;[2] Department of Chemical Engineering, Carnegie Mellon University, Pittsburgh, PA; [3]California Institute for Quantitative Biosciences (QB3), University of California, Berkeley, Berkeley, CA; [4]Picower Institute for Learning and Memory, Massachusetts Institute of Technology, Cambridge, MA; [5]Department of Brain and Cognitive Sciences, Massachusetts Institute of Technology, Cambridge, MA; [6]Department of Biomedical Engineering, Carnegie Mellon University, Pittsburgh, PA; [7]Department of Department of Neuroscience, University of California, Berkeley

Corresponding author emails: rpinals@mit.edu; kawhite@cmu.edu; landry@berkeley.edu

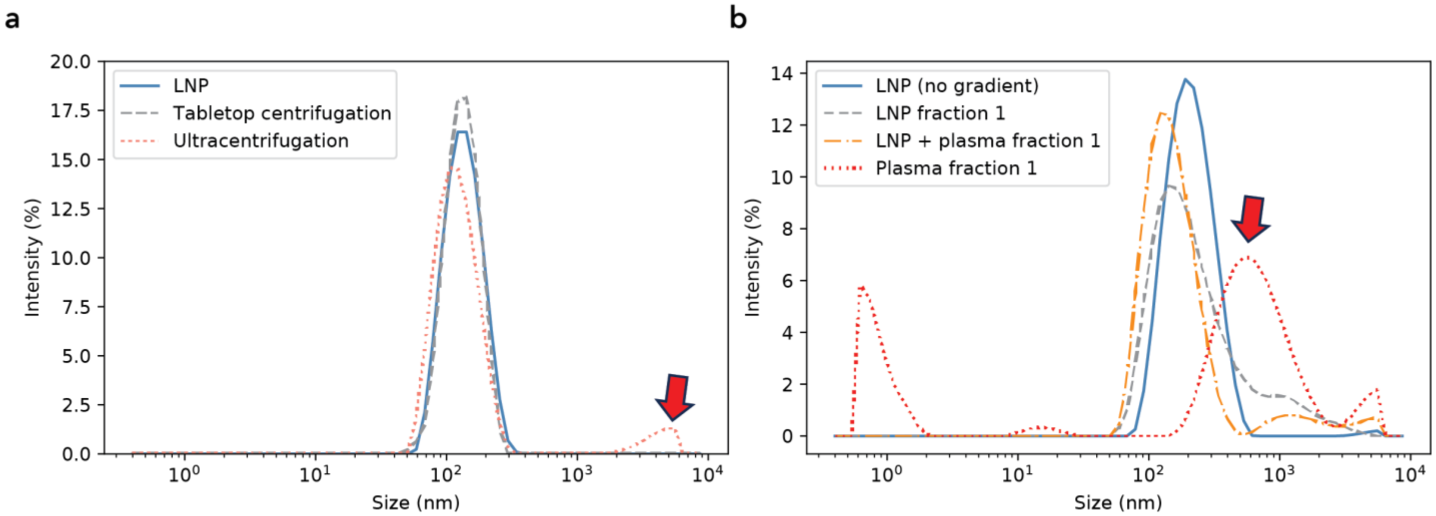

Supplementary Figure 1. DLS shows limitations of current techniques to isolate the protein corona on lipid nanoparticles (LNPs). (a) DLS of LNPs in the supernatant before (blue line) and after tabletop centrifugation (dotted grey line) and ultracentrifugation (dotted orange line) reveal lack of pelleting and aggregation (highlighted by red arrow), respectively (n = 3 technical replicates). (b) 1-mL fractions are collected top to bottom from prepared three-layer (30%, 15%, 0%) iodixanol density gradients of LNPs alone, LNPs incubated with plasma, and plasma alone centrifuged for 3 hours. DLS of current strategies for gradient layering for ultracentrifugation show that the native biological particles in the plasma control gradient (red arrow) are present in the parallel first fraction for the LNP gradient (n = 3 technical replicates).

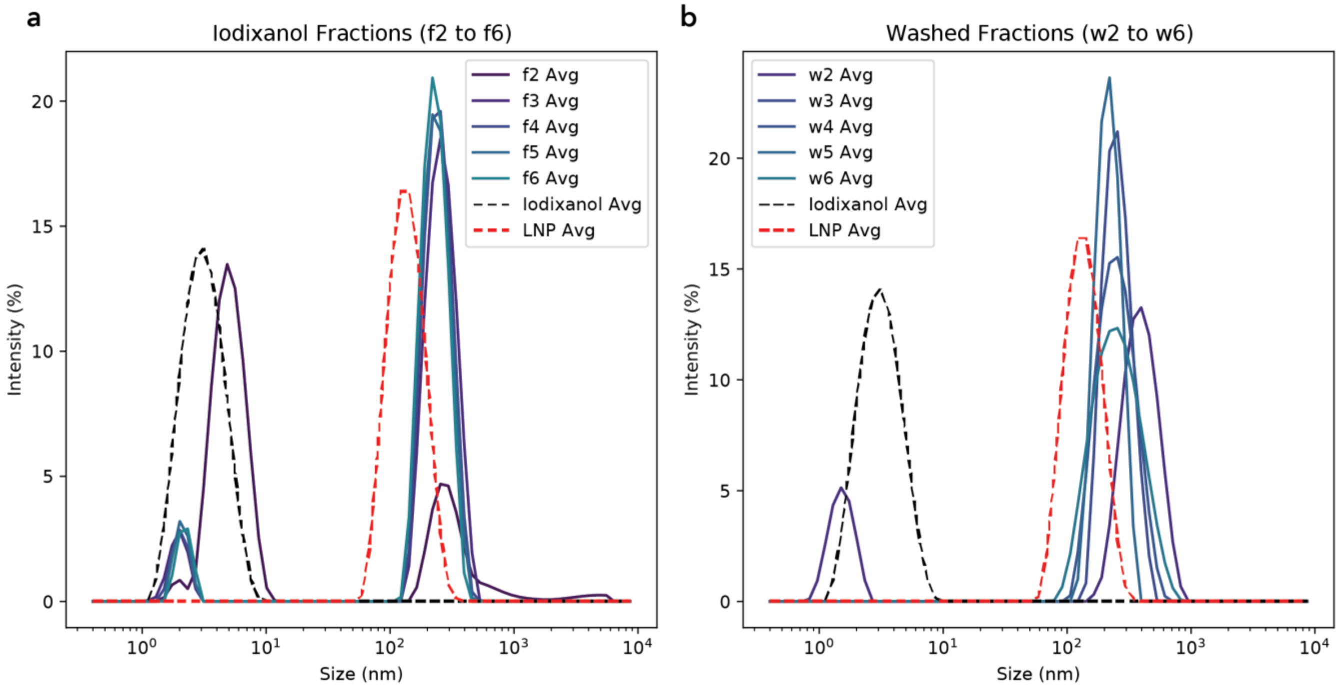

Supplementary Figure 2. DLS of particles after density gradient centrifugation (DGC). The LNPs were loaded into a density gradient and fractions were collected top to bottom according to the method used for proteomics characterization. To assess the stability of particles after centrifugation, LNP size was characterized via DLS in (a) the iodixanol medium and (b) after washing (n = 3 technical replicates). The fractions show similar sized LNPs to the control LNPs that did not undergo DGC (in red) with an additional peak present where iodixanol forms small particles in solution (dotted black line). The shift in size to the right is likely due to differences in solution properties. These measurements can only qualitatively confirm particle preservation because the refractive index and viscosity of the iodixanol changes within the gradient, affecting the DLS size calculations. The DLS calculation relies on the assumption that the particles are in a solution with the same refractive index and viscosity for comparison. To address this problem, we washed samples selected for proteomics characterization twice in PBS in 3-kDa MWCO Amicon centrifugal filters to remove the iodixanol medium. The washed particles also show similar size profiles to the control LNPs in red.

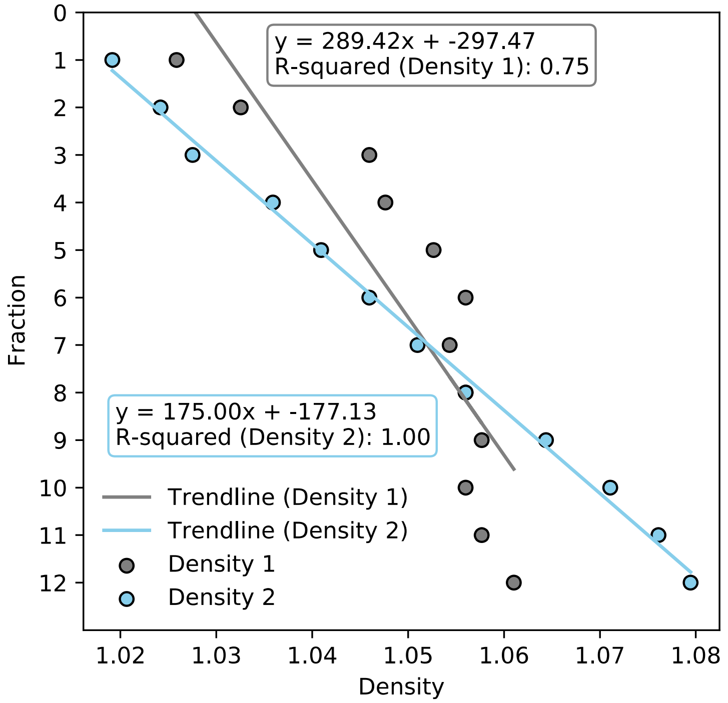

Supplementary Figure 3. Density linearity within the gradient. The density is calculated by measuring refractive index via refractometer and converting based on known standard curves for iodixanol. The density change within the first 12 fractions of the gradient must be linear to ensure appropriate separations. As shown above, bumping during gradient loading may result in a poor linear trend (gray line; Density 1, $R^2 = 0.75$). In contrast, careful layering will result in a linear density gradient (blue line; Density 2, $R^2 = 1.00$). These measurements highlight the need for quality control during gradient loading. Loading these gradients is technically challenging and overlooking quality control in density distribution throughout the tube may lead to LNP sample contamination and therefore misleading protein corona results.

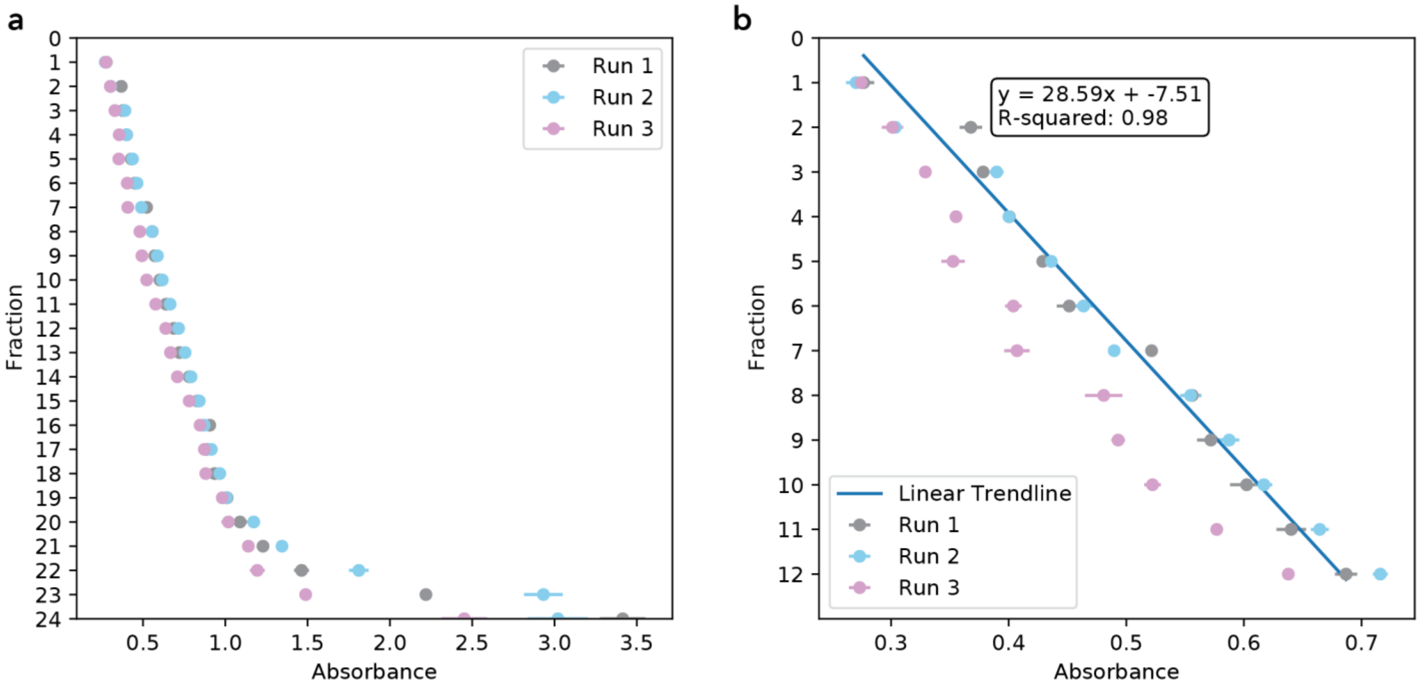

Supplementary Figure 4. Absorbance linearity within the gradient. (a) The absorbance at wavelength 340 nm, the peak absorbance for OptiPrep, was measured for fractions after DGC (n = 3 technical replicates). (b) As shown by the inset, the absorbance shows a linear trend within the fractions of interest. These absorbance measurements are a quick method for confirming successful gradient preparation, such as Run 2 with a linear trendline ($R^2 = 0.99$).

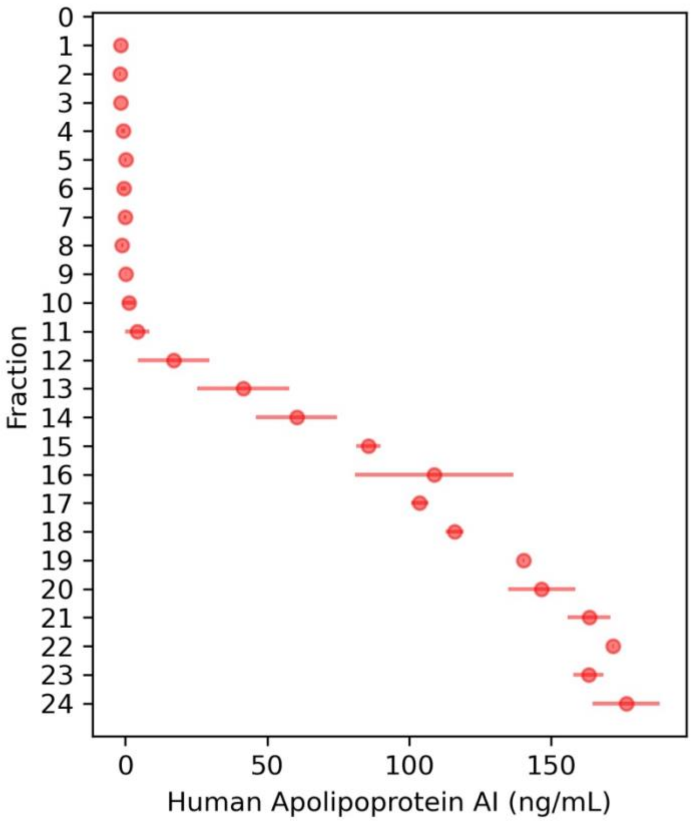

Supplementary Figure 5. Apolipoprotein AI localization within the density gradient. Average human apolipoprotein AI enzyme-linked immunosorbent assay quantification of plasma alone gradient fractions collected after DGC isolation workflow shows that 99.5% of apolipoprotein AI proteins are present among fractions 12-24 (n = 2 biological replicates). We estimated the percentage of lipoprotein A within the peak by calculating the area under the measured fluorescence curve for fractions 12-24 relative to the total area under the measured fluorescence curve using the trapezoidal rule (trapz function from scipy.integrate). Error bars all denote standard deviation.

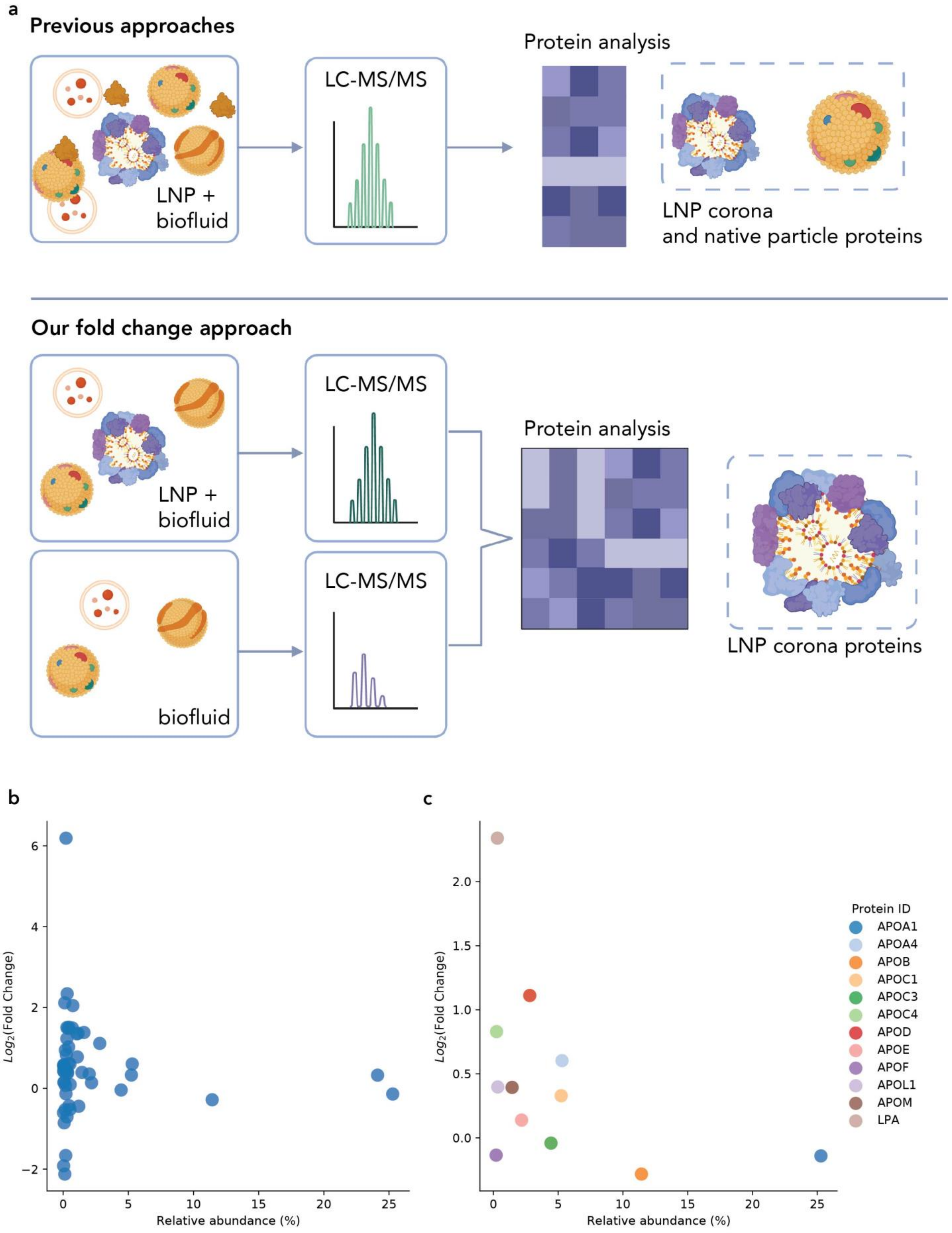
a
Previous approaches
LNP + biofluid
LC-MS/MS
Protein analysis
LNP corona and native particle proteins
Our fold change approach
LNP + biofluid
biofluid
LC-MS/MS
LC-MS/MS
Protein analysis
LNP corona proteins
b
Log_2(Fold Change)
Relative abundance (%)
c
Log_2(Fold Change)
Relative abundance (%)
Protein ID
APOA1
APOA4
APOB
APOC1
APOC3
APOC4
APOD
APOE
APOF
APOL1
APOM
LPA

Supplementary Figure 6. Comparison of logarithmic fold-change against relative abundance (%) for identified lipoproteins. (a) A comparison of previous approaches which examine relative abundance of only the LNP sample and our approach which quantifies differences between the LNP sample and a biofluid control. Voke, E. (2025) https://BioRender.com/58u4s4t. The scatter plots show the correlation between the data analysis workflow (fold change relative to plasma) suggested in this paper and previous approaches (relative abundance (%)) for (b) all identified proteins and (c) apolipoproteins (n = 3 technical replicates). The Pearson correlation coefficient for all proteins and lipoproteins was calculated to be -0.0871 and -0.426, respectively. The negative correlation between the two approaches highlights the need for more controlled approaches to analyzing the lipoprotein-LNP interactions.

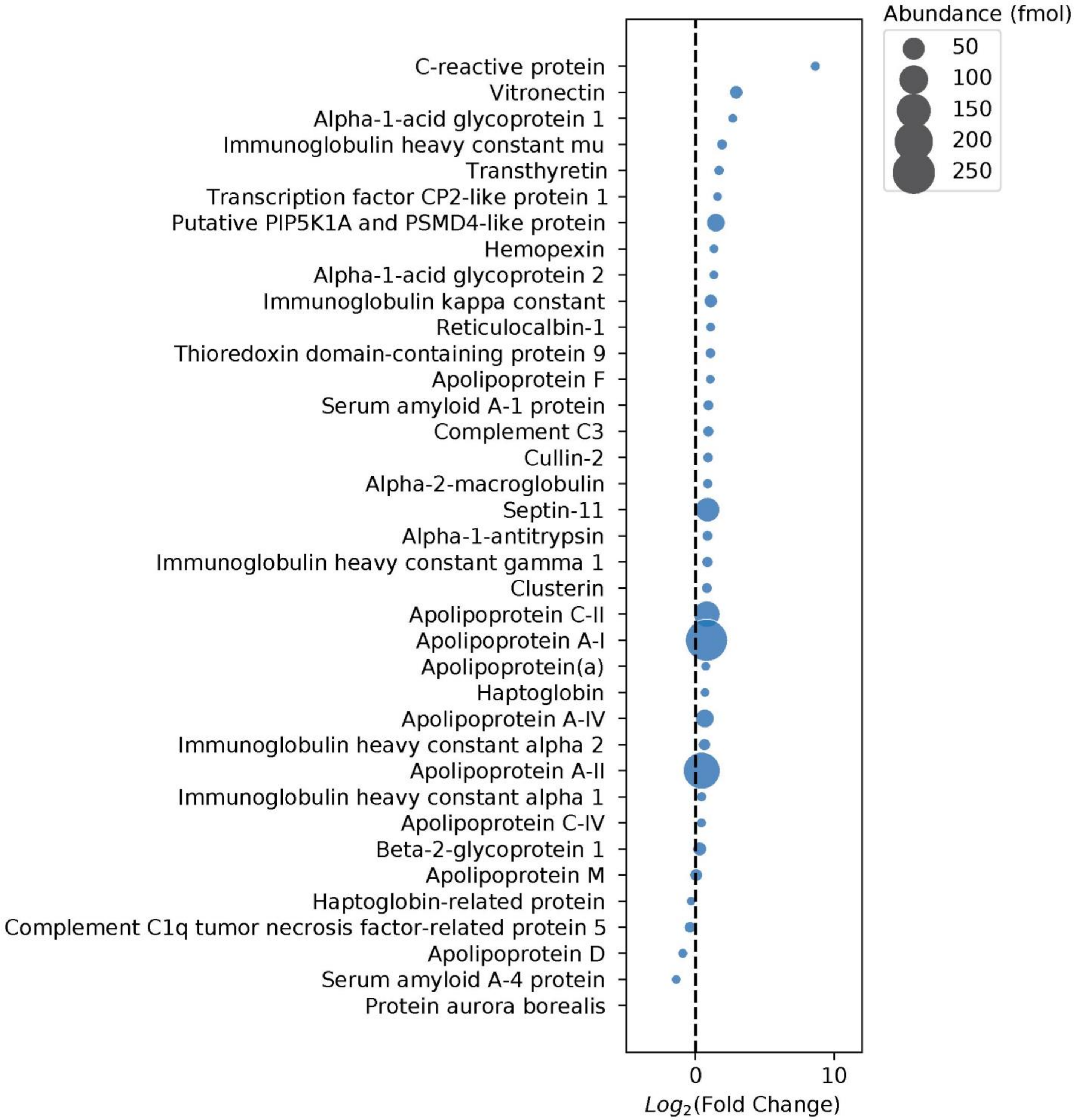

Supplementary Figure 7. LC-MS/MS of samples processed in parallel. Logarithmic fold change of proteins, with bubble size showing abundance (fmol) of samples processed in parallel (n = 3 biological replicates). The protein abundance for protein-LNP samples was analyzed relative to plasma control fractions and was filtered for adjusted p-value (q-value) <0.05. C-reactive protein and vitronectin were found to have high enrichment, in agreement with other datasets we collected. However, ApoE is not enriched across the parallel experiments, despite prior reports suggesting that ApoE adsorption drives downstream behavior including cell uptake.[1] Independent LC-MS/MS processing experiments show that differences in ApoE abundance (fmol) between the control sample and the LNP are not statistically different.

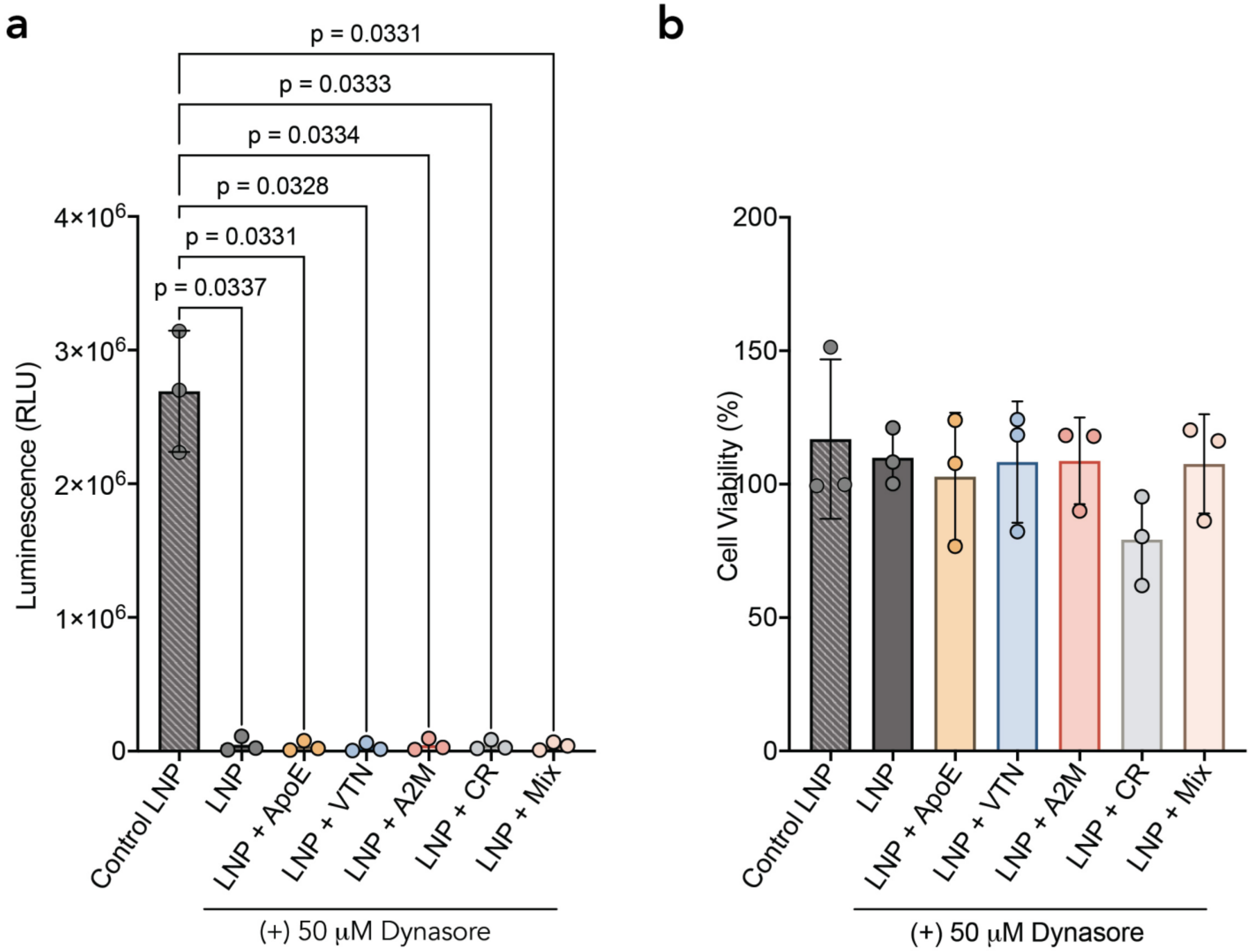

Supplementary Figure 8. Endocytosis inhibition of HepG2 cells incubated with protein-LNP complexes. LNPs loaded with mRNA encoding luciferase were incubated with selected high-binding corona proteins (0.05 ng mRNA : 1 ng protein) prior to introduction to HepG2 cells seeded at 4.7×10^4 cells per cm^2 (100 ng mRNA per well) pre-incubated for 30 minutes with 50 μM Dynasore endocytosis inhibitor. The luminescence was measured as a proxy for mRNA expression to understand the effect of proteins on LNP delivery efficiency in the presence of endocytosis inhibition. (a) Resulting luminescence of pre-incubations of individual proteins with LNPs showed a significant decrease in luminescence (mRNA expression) for all conditions with the inhibitor. (b) Cell viability showed no statistical difference for inhibitor incubations. N = 4 technical replicates, n = 3 biological replicates. Data points shown are biological replicates. Error bars all denote standard deviation. Statistical analysis was performed by one-way ANOVA test followed by Dunnett's multiple comparisons test.

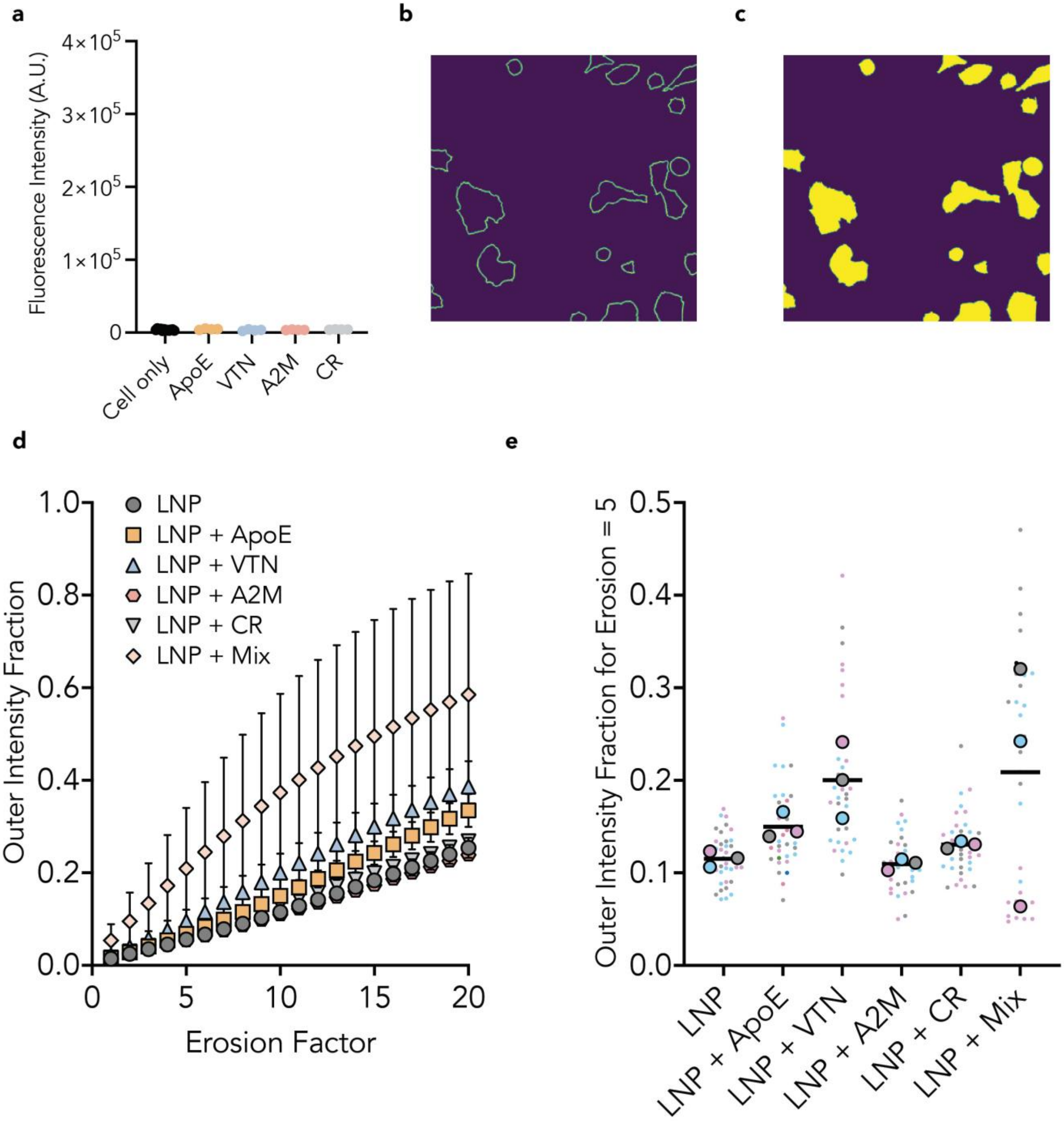

Supplementary Figure 9. Cy5 signal cellular localization analysis. (a) Cy5 fluorescence intensity per cell of cells incubated with protein show no significant signal. To compare the position of the LNPs associated with the cell (n = 4 technical replicates), (b) the signal in the outer regions of the cells shown in green relative to (c) the rest of the cell membrane shown in yellow was analyzed. (d) To compare the position of the LNPs associated with the cell, the signal in the outer regions of the cells relative to the rest of the cell membrane was analyzed across different levels of erosion. Localization analysis reveals that the VTN- and Mix-LNP complexes had generally more signal in the outer region of the cell in comparison to LNPs alone. (e) These changes in outer intensity are observed at erosion = 5, depicted in (b) where each dot represents an individual scene-level measurement, color-coded by biological replicate; black-outlined dots show biological replicate means. No statistical difference was observed. N = 4 technical replicates, n = 3 biological replicates. Data points shown are 3 averaged FOV for each technical replicate. Error bars all denote standard deviation. Statistical analysis was performed by Brown-Forsythe and Welch ANOVA test, followed by Dunnett's multiple comparisons test.

a

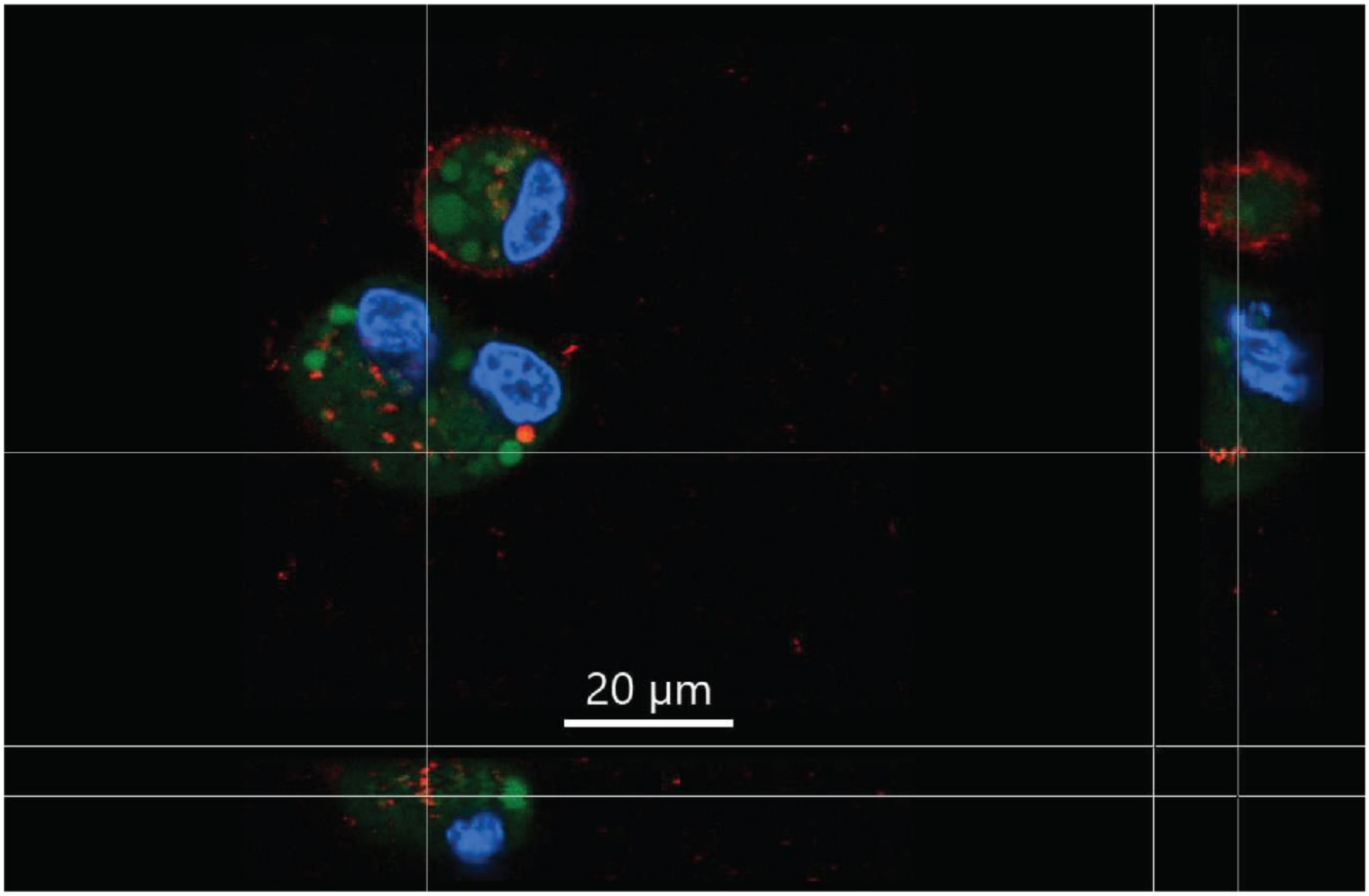

b

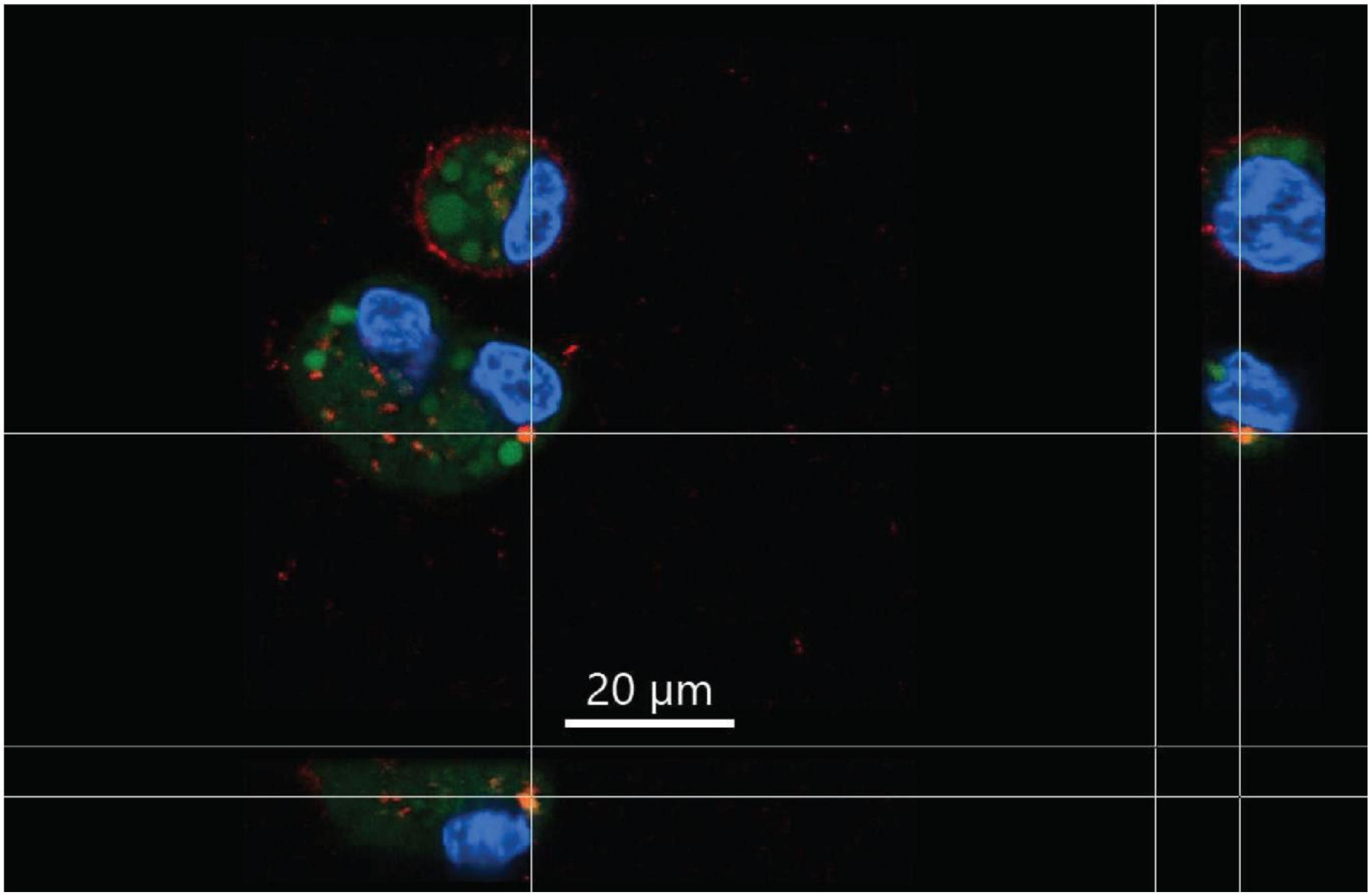

Supplementary Figure 10. Lysosomal co-localization of protein-LNP complexes in HepG2 cells. (a-b) HepG2 cells internalizing LNPs loaded with Cy5-mRNA incubated with VTN corona proteins were visualized by confocal microscopy. LNPs labeled with Cy5 (red), lysosomes (green), and nuclei (blue) were fluorescently imaged. Imaris-rendered cross-section view of Z-stack at 4.5 μm offset from bottom of cells illustrates that the LNPs are within the cell. A total of 50 Z-slices spanning a 20 μm depth from the bottom to the top of the cell were collected for cross-sectional rendering.

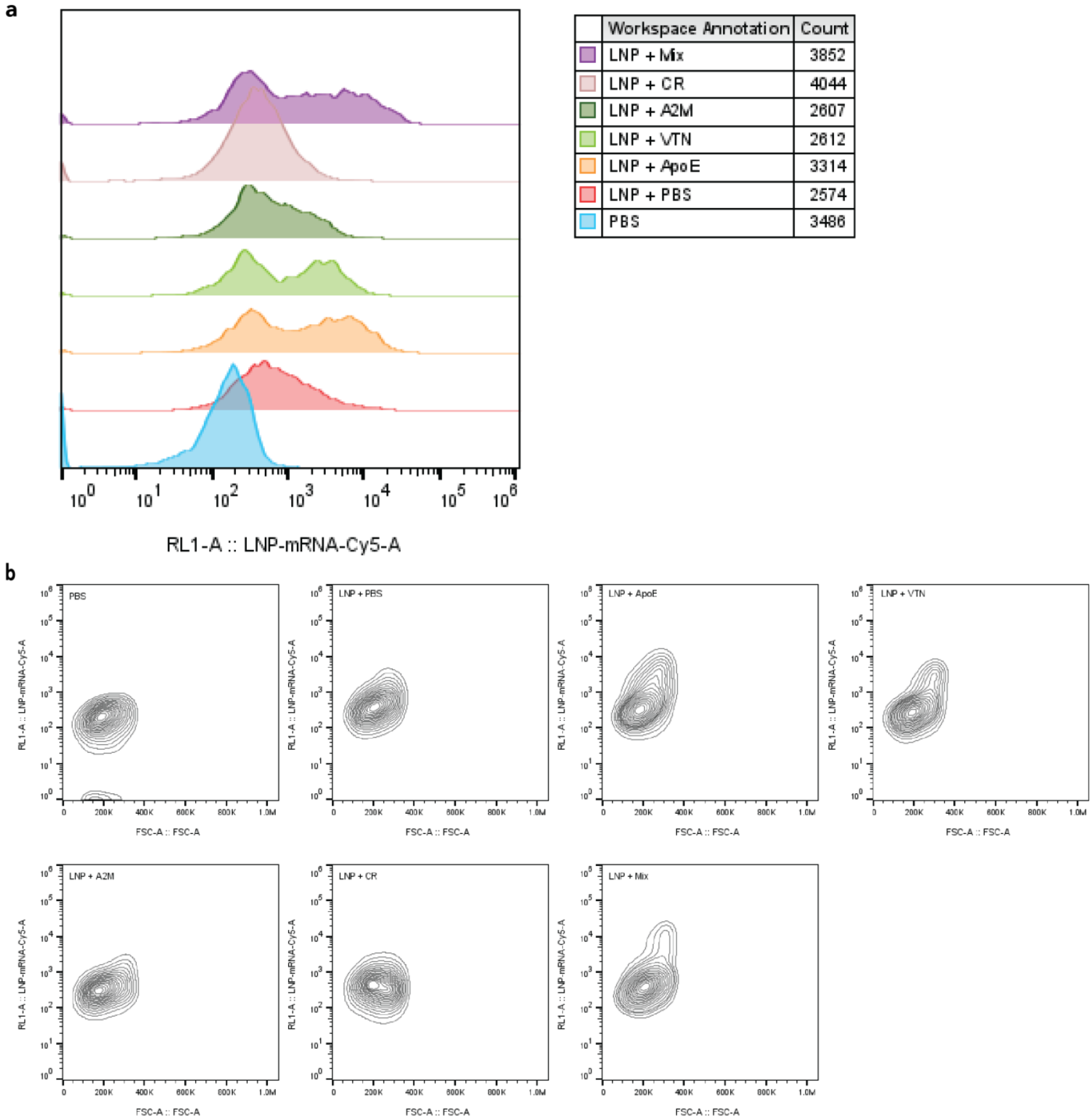

Supplementary Figure 11. Extended flow cytometry analysis for HepG2 uptake of protein–LNP complexes. (a) Representative histogram overlay showing fluorescence intensity distributions of HepG2 cells treated with protein–LNP formulations, corresponding to conditions shown in Figure 5. Fluorescence corresponds to Cy5-labeled mRNA cargo and cell counts are annotated. (b) Corresponding contour density plots illustrating gated single-cell populations across forward scattering. These plots support the quantification in Figure 5 by visualizing the distribution and separation of Cy5-mRNA–positive populations.

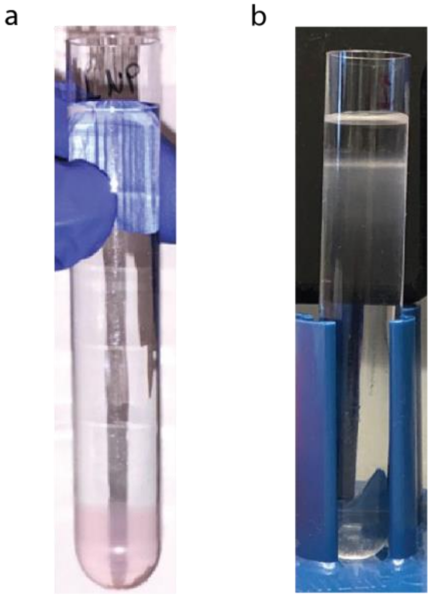

Supplementary Figure 12. Linearity within the gradient. (a) The gradient should be loaded such that the layers are visually distinguishable due to their differences in refractive index. The LNPs (here tagged with a dye for visualization) are loaded on the bottom of the gradient. (b) After centrifugation, the LNPs (undyed) should partition throughout the tube according to their density.

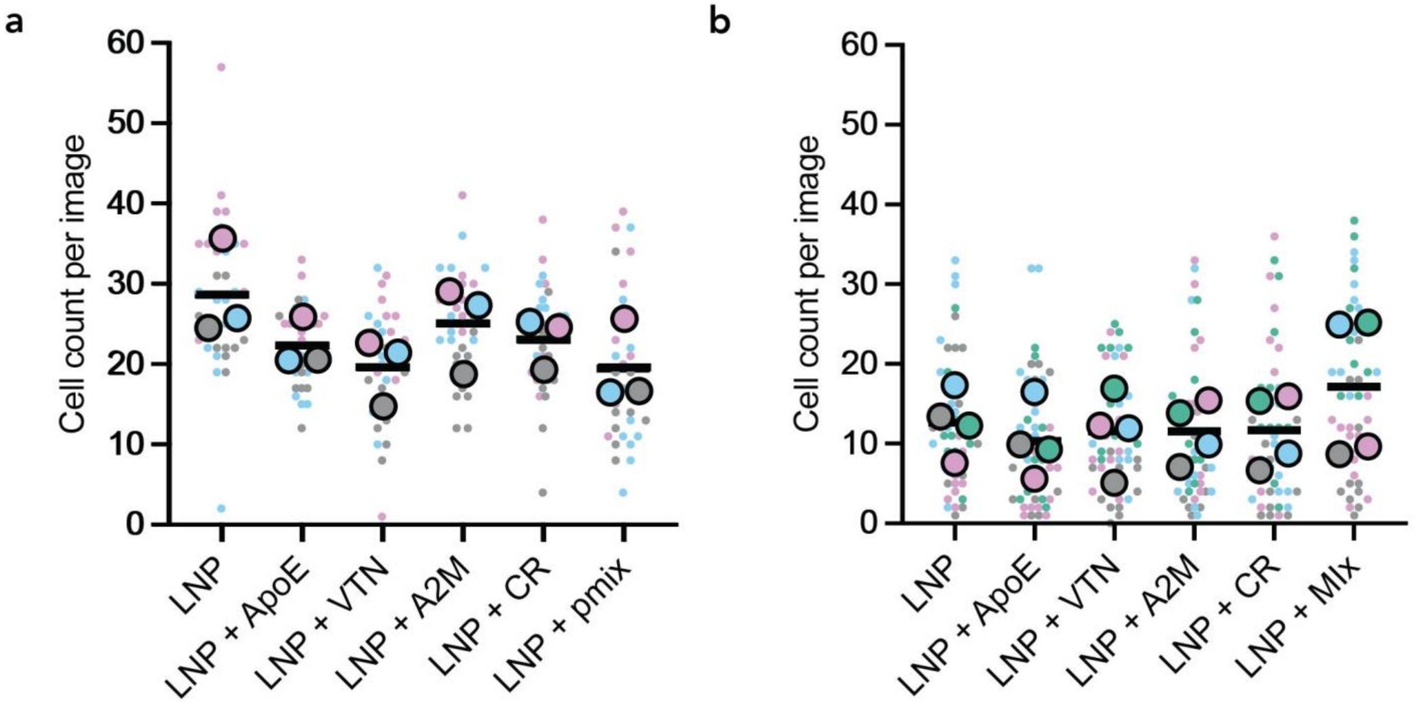

Supplementary Figure 13. Average cell count per image for confocal microscopy experiments. (a) Cell count per image for HepG2 cells internalizing LNPs loaded with Cy5-mRNA incubated with high-binding corona proteins. Across all conditions an average of 23 cells per image were detected. No significant differences were observed between LNPs pre-incubated with and without proteins. N = 4 technical replicates, n = 3 biological replicates. (b) Cell count per image for lysosomal colocalization analysis. Across all conditions an average of 12.5 cells per image were detected. No significant differences between LNPs pre-incubated with or without proteins. N = 4 technical replicates, n = 3 biological replicates. Each dot represents an individual scene-level measurement, color-coded by biological replicate; black-outlined dots show biological replicate means. Statistical analysis was performed by a nested one-way ANOVA test followed by Dunnett's multiple comparisons test.

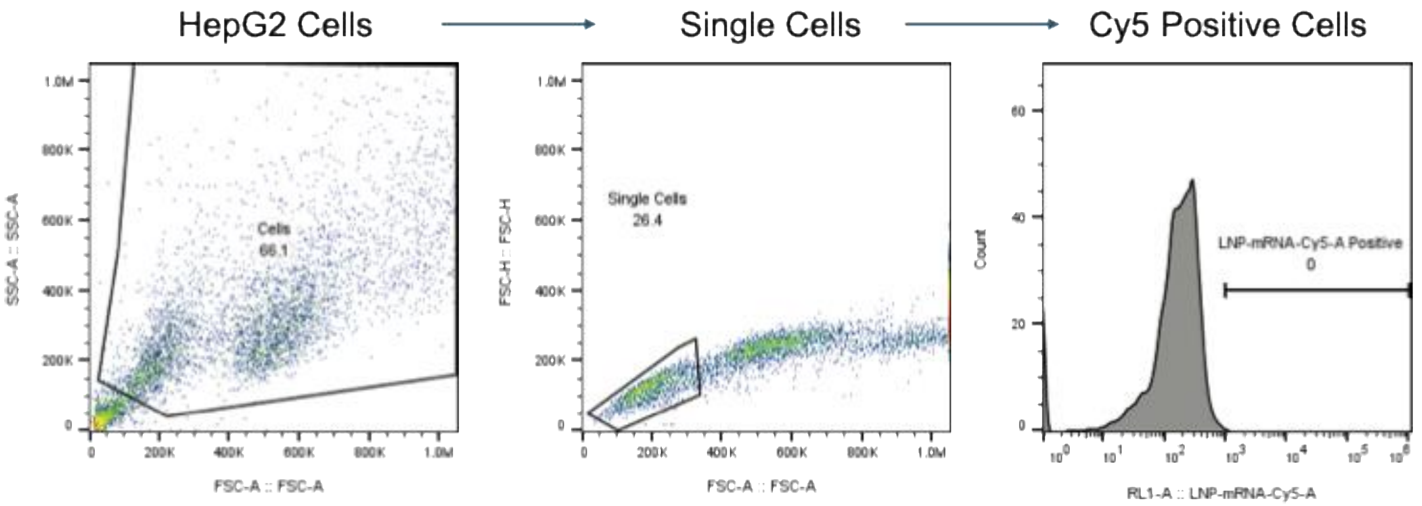

Supplementary Figure 14. Representative gating strategy for Cy5-mRNA detection by flow cytometry. Flow cytometry scatterplots and histograms showing a representative gating strategy for Figure 5. Single cells were first identified by FSC-A vs. FSC-H gating. Next, the Cy-5 mRNA fluorescence was analyzed, with the Cy-5 mRNA positive gate set based on a control of non-treated cells.

Supplementary Table 1. LNP localization within the density gradient.

Experiment #	Area under the curve (%)
Run 1	48.62
Run 2	72.25
Run 3	81.70

We estimated the percentage of LNPs within the peak by calculating the area under the measured fluorescence curve (Fig. 2c) for each run between fractions 2-6 relative to the total area under the measured fluorescence curve using the trapezoidal rule (trapz function from scipy.integrate). A baseline area, connected by the first and last data points, is subtracted to adjust the baseline to zero. The average was determined to be 67.52 with a standard deviation of 13.91.

Supplementary Table 2. Relative abundance of serum albumin in upper fractions of density gradient.

	**Serum albumin relative abundance (%)**	
Method	*LNP sample*	*Plasma control sample*
**Method 1**<br>4 hour, 40 k rpm, 4 °C, layers of 15% and 30% iodixanol	90.88	70.26
**Method 2**<br>16 hour, 36 k rpm, 4 °C, layers of 30%, 25%, 20%, 15%, 10%, and 5% iodixanol	24.12	21.22

Supplementary Table 2 shows the decrease in relative corona abundance for the most blood plasma-abundant protein, serum albumin. This suggests an overall improvement in the separation of free proteins from the fractions containing the LNPs. The relative abundance for the method 1 was calculated by determining the average relative abundance across fractions 2-6, which is the equivalent of pooled fractions 2-6 in method 2.

Supplementary Table 3. Proteins enriched across three independent experiments.

Protein	Log_2(fold change)	STDV
Alpha-2-macroglobulin	1.31	0.42
Apolipoprotein(a)	2.52	0.50
C-reactive protein	8.47	2.07
Haptoglobin-related protein	1.09	0.62
Vitronectin	2.33	0.25

Supplementary Table 4. Estimate of protein mass relative to mRNA mass based on LC-MS/MS abundances.

Protein	Abundance detected via LC-MS/MS (fmol)	MW (g/mol)	Mass of protein (ng)	Ratio of mRNA to protein
**CRP**	4.04	25039	0.10	1.98E+05
**VTN**	13.74	54306	0.75	2.68E+04
**A2M**	5.25	163291	0.86	2.33E+04
**ApoE**	40.25	36154	1.46	1.37E+04

Supplementary Table 5. Estimate of protein concentrations for selected proteins in native human plasma.

Protein	Native human plasma concentration (mg/mL)
**CRP**	0.009 (ref)[2]
**VTN**	0.02 (ref)[3]
**A2M**	178 (ref)[4]
**ApoE**	0.05 (ref)[5]

Note: The total concentration of protein in solution for protein incubations was 0.01 mg/mL (*2 µg in 200 µL of media*).

Supplementary Table 6. Dynamic light scattering intensity mean size and polydispersity index (PDI) of LNPs incubated with proteins for 1 hour at 37 °C (n = 3 technical replicates).

	Intensity Mean Size (nm)	Intensity Mean Size STDV	PDI	PDI STDV
**LNP**	129	1.9	0.101	0.005
**LNP + ApoE**	139	3.1	0.107	0.027
**LNP + VTN**	136	1.2	0.116	0.006
**LNP + A2M**	142	7.2	0.171	0.030
**LNP + CR**	188	0.8	0.094	0.002
**LNP + Mix**	168	29.3	0.167	0.016

References

(1) Akinc, A.; Querbes, W.; De, S.; Qin, J.; Frank-Kamenetsky, M.; Jayaprakash, K. N.; Jayaraman, M.; Rajeev, K. G.; Cantley, W. L.; Dorkin, J. R.; Butler, J. S.; Qin, L.; Racie, T.; Sprague, A.; Fava, E.; Zeigerer, A.; Hope, M. J.; Zerial, M.; Sah, D. W.; Fitzgerald, K.; Tracy, M. A.; Manoharan, M.; Koteliansky, V.; Fougerolles, A. de; Maier, M. A. Targeted Delivery of RNAi Therapeutics With Endogenous and Exogenous Ligand-Based Mechanisms. *Molecular Therapy* **2010**, *18* (7), 1357–1364. https://doi.org/10.1038/mt.2010.85.

(2) *C-Reactive Protein (CRP) Test: What It Is, Purpose & Results*. Cleveland Clinic. https://my.clevelandclinic.org/health/diagnostics/23056-c-reactive-protein-crp-test (accessed 2024-02-03).

(3) Clemetson, K. J. Chapter 9 - Blood glycoproteins⁎*~~~~This Chapter Is Dedicated to Prof. R.U. Lemieux Who Played a Major Role in Awakening a Whole Generation to the Importance of Carbohydrate Structure in Biology. In *New Comprehensive Biochemistry*; Montreuil, J., Vliegenthart, J. F. G., Schachter, H., Eds.; Glycoproteins II; Elsevier, 1997; Vol. 29, pp 173–201. https://doi.org/10.1016/S0167-7306(08)60622-5.

(4) Mocchegiani, E.; Giacconi, R.; Muti, E.; Muzzioli, M.; Cipriano, C. Zinc-Binding Proteins (Metallothionein and α-2 Macroglobulin) as Potential Biological Markers of Immunosenescence. In *NeuroImmune Biology*; Straub, R. H., Mocchegiani, E., Eds.; The Neuroendocrine Immune Network in Ageing; Elsevier, 2004; Vol. 4, pp 23–40. https://doi.org/10.1016/S1567-7443(04)80004-8.

(5) Mahley, R. W.; Innerarity, T. L.; Rall, S. C.; Weisgraber, K. H. Plasma Lipoproteins: Apolipoprotein Structure and Function. *Journal of Lipid Research* **1984**, *25* (12), 1277–1294. https://doi.org/10.1016/S0022-2275(20)34443-6.
