# Supplementary material for: Protein corona formed on lipid nanoparticles compromises delivery efficiency of mRNA cargo
Source: Nat Commun. 2025 Sep 30;16:8699. doi: 10.1038/s41467-025-63726-2 (PMC12485112; doi:10.1038/s41467-025-63726-2)
Supplement: Supplementary file 2 — Reporting summary [file 41467_2025_63726_MOESM2_ESM.pdf]

Corresponding author(s): Rebecca L. Pinals, Kathryn A. Whitehead, and Markita Landry

Last updated by author(s): 07/21/2025

Reporting Summary

Nature Portfolio wishes to improve the reproducibility of the work that we publish. This form provides structure for consistency and transparency in reporting. For further information on Nature Portfolio policies, see our Editorial Policies and the Editorial Policy Checklist.

Statistics

For all statistical analyses, confirm that the following items are present in the figure legend, table legend, main text, or Methods section.

n/a	Confirmed	
☐	☒	The exact sample size (n) for each experimental group/condition, given as a discrete number and unit of measurement
☐	☒	A statement on whether measurements were taken from distinct samples or whether the same sample was measured repeatedly
☐	☒	The statistical test(s) used AND whether they are one- or two-sided<br>*Only common tests should be described solely by name; describe more complex techniques in the Methods section.*
☒	☐	A description of all covariates tested
☐	☒	A description of any assumptions or corrections, such as tests of normality and adjustment for multiple comparisons
☐	☒	A full description of the statistical parameters including central tendency (e.g. means) or other basic estimates (e.g. regression coefficient) AND variation (e.g. standard deviation) or associated estimates of uncertainty (e.g. confidence intervals)
☐	☒	For null hypothesis testing, the test statistic (e.g. F, t, r) with confidence intervals, effect sizes, degrees of freedom and P value noted<br>*Give P values as exact values whenever suitable.*
☒	☐	For Bayesian analysis, information on the choice of priors and Markov chain Monte Carlo settings
☒	☐	For hierarchical and complex designs, identification of the appropriate level for tests and full reporting of outcomes
☒	☐	Estimates of effect sizes (e.g. Cohen's d, Pearson's r), indicating how they were calculated

Our web collection on statistics for biologists contains articles on many of the points above.

Software and code

Policy information about availability of computer code

Data collection	Peptide and protein identification and quantification using a label-free approach were performed using Progenesis QI for Proteomics software (version 4.2, Waters Nonlinear Dynamics). Zen Blue 3.2 software was used for imaging data collection. Attune NxT Software was used for flow cytometry.
Data analysis	Statistical analysis and visualization were performed with GraphPad Prism (v.10.2.3) and Python (v3). Further detailed imaging analysis is available (https://github.com/tengjuilin/internalization-analysis). Imaris-rendered images are included in the Supplemental Information.

For manuscripts utilizing custom algorithms or software that are central to the research but not yet described in published literature, software must be made available to editors and reviewers. We strongly encourage code deposition in a community repository (e.g. GitHub). See the Nature Portfolio guidelines for submitting code & software for further information.

Data

Policy information about availability of data

All manuscripts must include a data availability statement. This statement should provide the following information, where applicable:

- Accession codes, unique identifiers, or web links for publicly available datasets
- A description of any restrictions on data availability
- For clinical datasets or third party data, please ensure that the statement adheres to our policy

Source data are provided with the paper. The proteomic datasets generated during and analyzed during the current study are available in the MassIVE repository,

[doi:10.25345/C5251FZ24, identifier: MSV000098245]. The additional data that support the findings of this study are available from the corresponding author upon reasonable request.

Research involving human participants, their data, or biological material

Policy information about studies with human participants or human data. See also policy information about sex, gender (identity/presentation), and sexual orientation and race, ethnicity and racism.

Reporting on sex and gender	Findings do not apply to one sex or gender. Sex and gender were not considered in the study. We used pooled human plasma derived from multiple patients for our study. We do not have information on sex or gender for this biological material.
Reporting on race, ethnicity, or other socially relevant groupings	Socially constructed categorization variables were not used in this manuscript.
Population characteristics	See above.
Recruitment	We did not have any human subjects.
Ethics oversight	We did not have any human subjects.

Note that full information on the approval of the study protocol must also be provided in the manuscript.

Field-specific reporting

Please select the one below that is the best fit for your research. If you are not sure, read the appropriate sections before making your selection.

☒ Life sciences ☐ Behavioural & social sciences ☐ Ecological, evolutionary & environmental sciences

For a reference copy of the document with all sections, see nature.com/documents/nr-reporting-summary-flat.pdf

Life sciences study design

All studies must disclose on these points even when the disclosure is negative.

Sample size	Sample sizes were based on standard practice in the field for in vitro assays, where n = 3–4 technical replicates and n = 3-4 biological replicates per condition is sufficient to observe reproducible effects and perform appropriate statistical testing.
Data exclusions	No data were excluded from the analyses. Data was filtered according to parameters outlined in the methods section.
Replication	All attempts at replication were successful.
Randomization	Randomization was not performed, as all samples were treated under identical conditions using a single cell line and pooled plasma.
Blinding	Blinding was not relevant as this study involved pooled human plasma and a single cell line, with outcomes measured using automated, quantitative assays.

Reporting for specific materials, systems and methods

We require information from authors about some types of materials, experimental systems and methods used in many studies. Here, indicate whether each material, system or method listed is relevant to your study. If you are not sure if a list item applies to your research, read the appropriate section before selecting a response.

Materials & experimental systems

n/a	Involved in the study	
☒	☐	Antibodies
☐	☒	Eukaryotic cell lines
☒	☐	Palaeontology and archaeology
☒	☐	Animals and other organisms
☒	☐	Clinical data
☒	☐	Dual use research of concern
☒	☐	Plants

Methods

n/a	Involved in the study	
☒	☐	ChIP-seq
☐	☒	Flow cytometry
☒	☐	MRI-based neuroimaging

Eukaryotic cell lines

Policy information about cell lines and Sex and Gender in Research

Cell line source(s)	UC Berkeley Cell Culture Facility which is supported by The University of California Berkeley.
Authentication	Cell lines were authenticated by the UC Berkeley Cell Culture Facility using short tandem repeat profiling using Promegas GenePrint 10 kit.
Mycoplasma contamination	Cell lines were tested by the UC Berkeley Cell Culture Facility for mycoplasma using a nuclear stain and a 100x lens objective.
Commonly misidentified lines (See ICLAC register)	Commonly misidentified cell lines were not used.

Plants

Seed stocks	We did not use any plants in this study.
Novel plant genotypes	We did not use any plants in this study.
Authentication	We did not use any plants in this study.

Flow Cytometry

Plots

Confirm that:

- [x] The axis labels state the marker and fluorochrome used (e.g. CD4-FITC).
- [x] The axis scales are clearly visible. Include numbers along axes only for bottom left plot of group (a 'group' is an analysis of identical markers).
- [x] All plots are contour plots with outliers or pseudocolor plots.
- [x] A numerical value for number of cells or percentage (with statistics) is provided.

Methodology

Sample preparation	The cells were seeded into a white-bottom 96-well plate at a density of 15,000 cells per well. After treatment and incubation, the cells were washed with PBS, removed from plates via trypsin digestion, and resuspended with FACS buffer (PBS, 2% FBS), then transferred to a round bottom 96-well plate.
Instrument	ThermoFisher Attune NxT Acoustic Focusing Cytometer (Thermo Fisher Scientific)
Software	FlowJo v10 (FlowJo Inc)
Cell population abundance	Around 3000 cells per sample, where the cells form a distinct population in the FSC/SSC plot.
Gating strategy	The Cy-5 mRNA positive gate is determined based on a control of PBS (No LNP) treated cells. As shown in Supplementary Figure 13, the gate is set above the autofluorescence of non-treated cells.

- [x] Tick this box to confirm that a figure exemplifying the gating strategy is provided in the Supplementary Information.
